# Supplementary material for: Effect of Trilobatin from Lithocarpus polystachyus Rehd on Gut Microbiota of Obese Rats Induced by a High-Fat Diet
Source: Nutrients. 2021 Mar 10;13(3):891. doi: 10.3390/nu13030891 (PMC8001797; doi:10.3390/nu13030891)
Supplement: Supplementary file 1 [file nutrients-13-00891-s001.pdf]

Supplementary information

Effect of Trilobatin from *Lithocarpus Polystachyus* Rehd on the Serum Lipid Levels and Gut Microbiota of Rats under High-fat Diet

Hailiang Shen[1,2†] , Linhua Huang[1,2†], Huating Dou[1,2,] , Yali Yang[3,4,*] , Houjiu Wu[1,2,*]

[1] Citrus Research Institute, Southwest University, Chongqing, P.R.China

[2] Citrus Research Institute, Chinese Academy of Agricultural Science, Chongqing, P.R. China

[3] Department of Food Engineering and Nutritional Science, Shaanxi Normal University, Xi'an, P.R. China

[4] National Research and Development Center of Apple processing Technology, P.R. China

Correspondence author at:

Southwest University, Citrus Research Institute, Beibei, Chongqing, 400712, P.R.China.

Tel: +86 023 68349701; Fax: +86 023 68349701. (Houjiu Wu)

Shaanxi Normal University, Department of Food Engineering and Nutritional Science, Campus Chang'an, No 620, West Chang'an Avenue, Chang'an District, Xi'an 710119, P.R.China. Tel: +86 029 85310471; Fax: +86 029 85310471. (Yali Yang)

E-mail address: wuhoujiu@cric.cn (Houjiu Wu), yangyali@snnu.edu.cn (Yali Yang)

[†]These authors contributed equally.

Table S1 the composition of chow diet

Compositions	Source	Value (g kg^{-1})
Protein	American chicken meal	Crude protein ≥ 180
		Lysine≥8
		Methionine + cysteine ≥ 5
Fat	Vegetable oil	Crude fat ≥ 40
Fiber	Bran	Crude fiber ≤ 50
Carbohydrate	Corn, wheat flour	-
Vitamin	Vitamin A, D, E, B and other multivitamins	-
Mineral	Iron, zinc and other complex trace elements	Calcium 15~18
		Total phosphorus 8~12

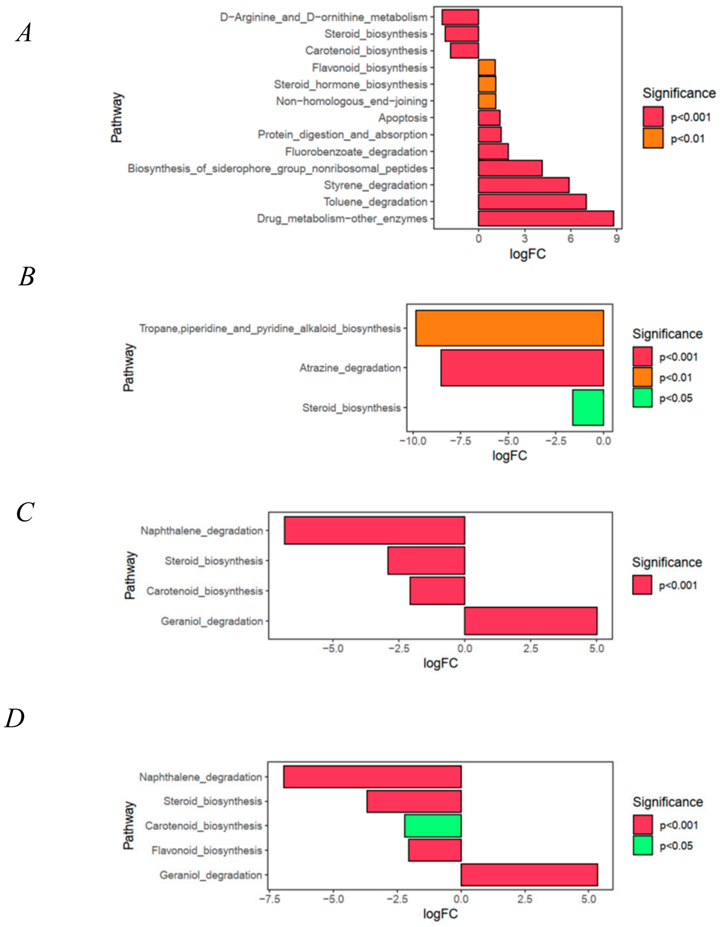

Fig. S1 KEGG metabolic pathways functional gene prediction analysis in different groups. (A) different KEGG metabolic pathways between NC and BC groups; (B) different KEGG metabolic pathways between BC and TRL groups; (C) different KEGG metabolic pathways between BC and TRM groups; (D) different KEGG metabolic pathways between BC and TRH groups. NC: Normal control group, chow diet; BC: Blank control group, High-fat diet; ORL: Orlistat; TRL: Low dose group, trilobatin; TRM: Middle dose group, trilobatin; TRH:High dose group, trilobatin.
